# Adverse and adaptive placental DNA methylation changes linking prenatal air pollution exposure to child lung function: findings from the SEPAGES cohort

**DOI:** 10.1016/j.ebiom.2026.106344

**Published:** 2026-06-24

**Authors:** Lucile Broséus, Valérie Siroux, Gaëlle Uzu, Anouk Marsal, Cécile Tassel, Jörg Tost, Anne Boudier, Stephan Gabet, Sofia Aguilar-Lacasaña, Frédérique White, Kyle A. Campbell, Todd M. Everson, Marie-France Hivert, Sylvain Carras, Sarah Lyon-Caen, Rémy Slama, Claire Philippat, Sam Bayat, Olivier François, Johanna Lepeule

**Affiliations:** a Université Grenoble Alpes, Inserm U1209, CNRS UMR 5309, Institut Pour l’Avancée des Biosciences (IAB), Team of Environmental Epidemiology Applied to Development and Respiratory Health, Grenoble, France; b Université Grenoble Alpes (UGA), Institut de Recherche pour le Développement (IRD), Centre National de la Recherche Scientifique (CNRS), INRAE, Grenoble INP, IGE (Institute of Environmental Geosciences), Grenoble, France; c Laboratory for Epigenetics and Environment, Centre National de Recherche en Génomique Humaine, CEA, Institut de Biologie François Jacob, University Paris Saclay, Evry, France; d Department of Pulmonology and Physiology, CHU Grenoble Alpes, Grenoble, France; e Univ. Lille, CHU Lille, Institut Pasteur de Lille, ULR 4483-IMPacts de l’Environnement Chimique sur la Santé (IMPECS), Lille, France; f ISGlobal, Barcelona, Spain; g Département de Biologie, Université de Sherbrooke, Sherbrooke, Quebec, Canada; h Gangarosa Department of Environmental Health, Emory University Rollins School of Public Health, Atlanta, GA, USA; i Department of Population Medicine, Harvard Medical School, Harvard Pilgrim Health Care Institute, Boston, MA, USA; j Biobank BB-0033-00069, Univ. Grenoble Alpes, Inserm U1209, CNRS UMR5309, Institute for Advanced Biosciences, CHU Grenoble-Alpes, Grenoble, France; k Université Grenoble Alpes, Inserm, UA07 STOBE Laboratory, Grenoble, France; l Université Grenoble Alpes, Centre National de la Recherche Scientifique (CNRS), Grenoble INP, TIMC CNRS UMR 5525, Grenoble, France; m Universitat Pompeu Fabra (UPF), Barcelona, Spain; n CIBER Epidemiología y Salud Pública, Madrid, Spain

**Keywords:** Air pollution, PM_2.5_ oxidative potential, DNA methylation, Placenta, Lung function, Mediation analysis

## Abstract

**Background:**

Prenatal air pollution exposure (PAPE) may have programming effects on offspring health. Placental epigenetic modifications are hypothesised to mediate this relationship, but evidence remains limited. We investigated the mediating role of placental DNA methylation (DNAm) in the association between prenatal exposure to ${\text{NO}}_{2}$, ${\text{PM}}_{2.5}$ and ${\text{PM}}_{2.5}$ oxidative potential (OP) and newborn lung function.

**Methods:**

Placental DNAm was measured using the Infinium HumanMethylationEPIC BeadChip in 395 participants from the French cohort SEPAGES. PAPE was estimated via personal sensors. Lung function (6 parameters) was measured at two months through tidal breathing analysis and nitrogen multiple breath washout test. We conducted adjusted epigenome-wide mediation analyses to identify genomic loci (CpGs and aggregated methylated regions; AMRs) that explain the association between PAPE and offspring lung function.

**Findings:**

Thirty CpGs and 166 AMRs significantly mediated the PAPE-lung function relationship. Most mediators were involved in the effect of ${PM}_{2.5}$ OP on the functional residual capacity (FRC) and the lung clearance index (LCI). While most DNAm changes mediated adverse effects, several loci—especially within imprinted genes—were associated with potentially adaptive responses. Mediating loci mapped to genes involved in lung development, immunity, inflammation and oxidative stress. Expression quantitative trait methylation (eQTM) analyses revealed that nearly one quarter of the mediating CpGs and one half of the AMRs may regulate gene expression.

**Interpretation:**

Our findings provide insights into the epigenetic pathways linking PAPE to early-life lung function. They highlight the placenta as a critical interface, where both detrimental and compensatory epigenetic modifications may shape newborn lung function.

**Funding:**

French Agency for National Research.

**Research in context**

**Evidence before this study**

Air pollution is a major environmental health problem, estimated to affect virtually everyone and to cause 4.2 million premature deaths worldwide, approximately one-third of which is attributable to respiratory diseases. Airborne particulate matter (PM) is a primary concern for respiratory health, notably due to its oxidative potential that reflects its ability to induce oxidative stress. Pregnancy is a period of increased susceptibility, and several systematic reviews have underlined the crucial role of air pollutants in shaping the long-term pulmonary health. Pregnant women are particularly exposed not only to outdoor air pollution but also to indoor air pollution, which can contain high levels of PM. However, few studies have relied on longitudinal assessments of personal exposure. Air pollution particles can cross the placental barrier, and placental epigenetic mechanisms are thought to contribute to both adverse and adaptive programming effects of maternal exposure on offspring lung function. We searched PubMed, from database inception to April 7th 2026, for publications that investigated the mediating role of placental DNA methylation (DNAm) in the relationship between prenatal air pollution exposure and child lung function. We used the terms: (“air pollution” OR “particulate matter” OR “oxidative potential”) AND (“lung” OR “lung function” OR “airway” OR “respiratory health”) AND (“prenatal” OR “pregnancy”) AND “DNA methylation” AND “placenta”. Although studies have highlighted associations between prenatal air pollutant exposure and alterations in placental DNA methylation patterns, none examined whether these epigenetic changes were related to child lung function. Moreover, all existing studies focused on ambient air pollution exposure and overlooked the influence of indoor exposure despite the importance to assess the total exposure of pregnant women. Furthermore, the effect of the oxidative potential of the PM on placental DNAm has not been explored.

**Added value of this study**

The study provides one of the most detailed assessments of maternal pregnancy exposure to air pollution and child lung function, combining personal monitoring of NO_2_, PM_2.5_ mass concentration and PM_2.5_ oxidative potential with innovative noninvasive measurements of six lung function parameters in newborns shortly after birth. Changes in placental DNA methylation patterns which explain (or mediate) the effect of maternal exposure to air pollution on child lung function were investigated thanks to a method tailored for epigenome-wide mediation analyses. Furthermore, the development of well-calibrated models, combined with site-specific and regional level explorations as well as gene expression data, allowed generating robust results.

We found evidence of mediation by placental DNAm for all lung function parameters at genes implicated in biological processes instrumental for the programming of child respiratory health, including lung development, immunity, inflammation and oxidative stress. Most air pollution-driven placental DNAm changes explained an adverse effect of maternal exposure to PM_2.5_ oxidative potential on newborn lung function. But a few DNAm modifications, notably located in imprinted genes, mediated a positive adaptive effect. Additional gene expression analyses highlighted that these epigenetic changes could have a functional impact.

**Implications of all the available evidence**

By identifying specific mediating placental epigenetic changes relevant to airway development and respiratory health, our findings strengthen existing evidence of a causal link between prenatal exposure to air pollution and newborn lung function. The placenta appears as a key mediator of early respiratory development and placental epigenetics can provide insights into early-life respiratory risks, emphasising the prenatal period as a relevant window for preventive interventions. Importantly, our findings emphasise the need to study these specific adaptive epigenetic changes more systematically, as these responses–which may help balance harmful effects–are too often neglected. They also underscore the importance of reducing pregnant women’s exposure to indoor and outdoor air pollution and considering the oxidative properties of PM when shaping public health policies.

## Introduction

Due to its ubiquity, air pollution remains a central concern for public health and is acknowledged to induce long-lasting effects on respiratory health.^1^ Lung development critically shapes long-term respiratory function, and early impairments often persist into adulthood with increased risks of premature mortality and chronic respiratory, cardiovascular and metabolic diseases.^2^ Owing to their developing physiology, fetuses’ lungs are particularly vulnerable to the effects of prenatal air pollution exposure (PAPE).^1^ PAPE has notably been found to be associated with a lower lung volume and with a higher respiratory need in children.^3^, ^4^, ^5^ The oxidative potential (OP) of particulate matter (PM), a measure of the ability of the inhaled particles to induce oxidative stress within the lung fluid and thus a part of the toxicity of the PM, has emerged as a relevant predictor of respiratory effects.^3^

PAPE may affect foetal lung function by remodelling the airway, influencing the programming of the immune system and triggering systemic inflammation and oxidative stress.^1^ PAPE may influence developmental plasticity by altering epigenetic patterns of the placenta, though the underlying molecular mechanisms remain largely unclear. The placenta plays a key role as a potential moderator and mediator of the effects of PAPE on offspring respiratory health.^1^^,^^6^ Maternal environmental stress can lead to specific adaptations and alterations in placental gene expression, partly regulated by epigenetic modifications such as DNA methylation (DNAm).^7^ Although PAPE-driven placental DNAm modifications have been observed in a wide range of biological pathways,^8^ their link to subsequent offspring lung function has not been explored yet.

PAPE-induced DNAm alterations may have several roles and interpretations. First, they could be markers of an incomplete buffering of toxic effects, when PAPE have exceeded the capacity for resilience. These modifications may then dysregulate specific placental functions, induce adverse effects on foetal development and underpin the predisposition to diseases later in life. Secondly, they may be markers of epigenetic adaptation, reflecting successful buffering responses to air pollutants to allow appropriate adaptation and maintain foetal development until birth. These placental adaptations may have persistent benefits, allowing the offspring to better fit and survive in an adverse environment, but they could also result in long-term hazards. Finally, some of these modifications can be neutral biomarkers of exposure.^6^^,^^9^ Recent progress in high-dimensional mediation analyses offers an efficient and valuable opportunity to elucidate the interpretation of exposure-induced placental DNAm changes.^10^^,^^11^ Mediation analysis allows to investigate the intermediate epigenetic mechanisms linking an exposure to a health outcome, by identifying significant exposure-induced DNAm modifications that have an effect on the outcome. An indirect (mediator) effect (IE) is estimated which reflects the effect of PAPE on lung function operating through each intermediate change in DNAm. The IE can then be interpreted to determine whether DNAm changes act as protective adaptation, adverse adaptation or incomplete buffering regarding the outcome of interest. Even in the absence of a statistically significant association between the exposure and the outcome, an exposure may trigger relevant intermediate biological mechanisms that affect health outcomes. These potential DNAm mediators are worth being investigated, because they can provide important insights both into the way an exposure affects the placenta and how the placenta protects against toxicity effects and further adapts to allow survival.^10^^,^^12^^,^^13^

This study investigates placental DNA modifications as a potential intermediate molecular mechanism linking maternal air pollutants and ${PM}_{2.5}$ oxidative potential exposure during pregnancy to newborn lung function. We took advantage of innovative noninvasive techniques to measure newborn lung function, personal exposure to air pollutants and ${\text{PM}}_{2.5}$ oxidative potential (OP) and of recent methodological advances in high-dimensional mediation analyses^11^ to identify PAPE-driven placental DNAm changes related to newborn lung function, and clarify the implications of these epigenetic alterations regarding early-life lung function. We then further investigated related biological pathways and their associations with gene expression to gain insight into the role and functional consequences of these modifications.

## Methods

### Study population

**Fig. 1:**
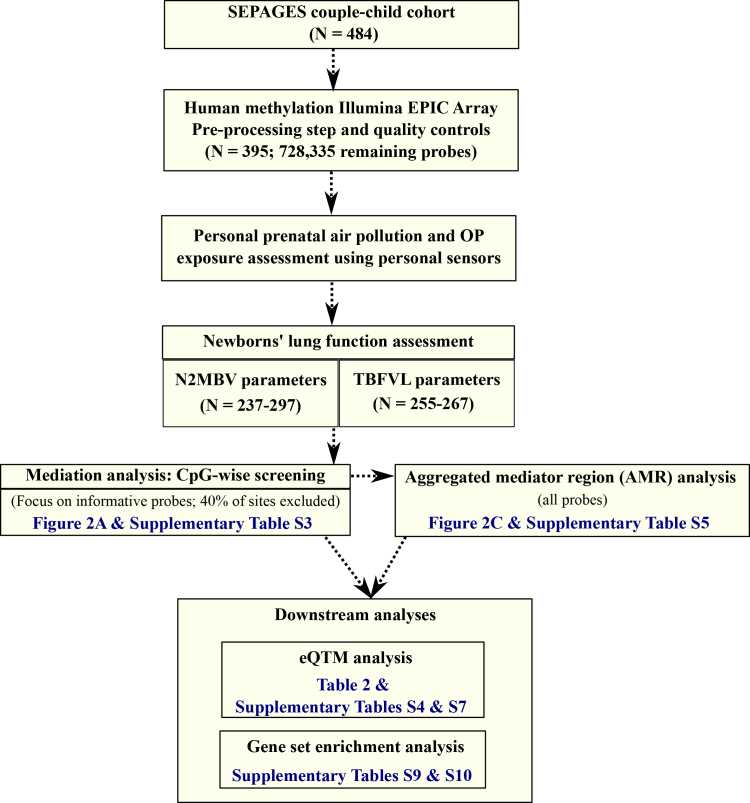
Workflow of the conducted analyses.

Our study relied on the French prospective couple-child cohort SEPAGES (Suivi de l’Exposition à la Pollution Atmosphérique durant la Grossesse et Effets sur la Santé).^14^ From 2014 to 2017, a total of 484 pregnant women were recruited in the Grenoble metropolitan area. The inclusion criteria were as follows: being pregnant by less than 19 gestational weeks, older than 18 years old, having a singleton pregnancy, being affiliated to the French national security system, planning to deliver in one of the four maternity clinics of Grenoble metropolitan area, and living in the study area. There were no pre-term births (gestational age < 37 weeks). Analyses were restricted to the 395 participants with available DNAm data (Fig. 1 and Table 1).

**Table 1:** Population characteristics.

	1. Whole cohort (N = 484)	2. Included in N2MBV analyses (N = 297)	3. Included in TBFVL analyses (N = 267)
Socio-demographic characteristics			
Child sex:			
Female	222 (45.9%)	135 (45.5%)	129 (48.3%)
Male	258 (53.3%)	162 (54.5%)	138 (51.7%)
‘Missing’	4 (0.83%)	0 (0.00%)	0 (0.00%)
Maternal age	32.1 [29.9; 35.2]	32.3 [29.9; 35.0]	32.1 [30.0; 35.2]
Parity:			
No child	222 (45.9%)	132 (44.4%)	114 (42.7%)
At least one child	262 (54.1%)	165 (55.6%)	153 (57.3%)
Maternal BMI	21.5 [19.8; 23.9]	21.5 [19.7; 23.7]	21.3 [19.7; 23.7]
Maternal smoking:			
Non smoker	384 (79.3%)	241 (81.1%)	222 (83.1%)
Smoker	55 (11.4%)	36 (12.1%)	34 (12.7%)
‘Missing’	45 (9.30%)	20 (6.73%)	11 (4.12%)
Parental rhinitis:			
No	164 (33.9%)	108 (36.4%)	102 (38.2%)
Yes	276 (57.0%)	174 (58.6%)	150 (56.2%)
‘Missing’	44 (9.09%)	15 (5.05%)	15 (5.62%)
Parental highest educational attainment:			
<MasterDegree	147 (30.4%)	86 (29.0%)	68 (25.5%)
≥MasterDegree	335 (69.2%)	209 (70.4%)	197 (73.8%)
‘Missing’	2 (0.41%)	2 (0.67%)	2 (0.75%)
EDI	−0.94 [−2.80; 0.92]	−0.86 [−2.70; 1.00]	−0.86 [−2.73; 0.79]
Season of conception:			
January–March	124 (25.6%)	82 (27.6%)	82 (30.7%)
April–June	103 (21.3%)	63 (21.2%)	57 (21.3%)
July–September	112 (23.1%)	61 (20.5%)	52 (19.5%)
October–December	145 (30.0%)	91 (30.6%)	76 (28.5%)
Gestational duration	40.0 [39.0; 40.0]	40.0 [39.0; 40.0]	40.0 [39.0; 40.0]
Delivery mode:			
C-section	81 (16.7%)	39 (13.1%)	34 (12.7%)
Vaginal	398 (82.2%)	258 (86.9%)	233 (87.3%)
‘Missing’	5 (1.03%)	0 (0.00%)	0 (0.00%)
Child length (2 months)	56.2 [55.0; 58.0]	56.2 [55.0; 58.0]	56.0 [55.0; 58.0]
Child weight (2 months)	4.79 [4.45; 5.22]	4.81 [4.47; 5.24]	4.80 [4.42; 5.22]
Child passive smoking			
No	329 (68.0%)	217 (73.1%)	200 (74.9%)
Yes	86 (17.8%)	56 (18.9%)	53 (19.9%)
‘Missing’	69 (14.3%)	24 (8.08%)	14 (5.24%)
Season of exam			
January–March	101 (20.9%)	72 (24.2%)	68 (25.5%)
April–June	91 (18.8%)	53 (17.8%)	54 (20.2%)
July–September	107 (22.1%)	77 (25.9%)	57 (21.3%)
October–December	133 (27.5%)	95 (32.0%)	88 (33.0%)
‘Missing’	52 (10.7%)	0 (0.00%)	0 (0.00%)
Average air pollution exposure during pregnancy			
${{\text{PM}}_{2.5}\;OP}^{\text{DTT}}$	1.50 [1.10; 2.00]	1.49 [1.05; 1.97]	1.49 [1.05; 1.97]
${\;{\text{PM}}_{2.5}\;OP}^{\text{AA}}$	1.56 [1.07; 2.24]	1.50 [1.03; 2.19]	1.56 [1.07; 2.24]
Personal ${\text{PM}}_{2.5}$	13.2 [10.3; 17.3]	13.2 [10.5; 17.4]	13.2 [10.4; 17.4]
Personal ${NO}_{2}$	19.2 [15.2; 24.0]	19.4 [15.3; 24.0]	19.4 [15.5; 23.8]
Ambient ${\text{PM}}_{2.5}$	13.5 [11.2; 16.1]	13.3 [10.9; 16.1]	13.4 [11.1; 16.2]
Ambient ${\text{PM}}_{10}$	20.2 [17.6; 22.6]	20.1 [17.3; 22.6]	20.0 [17.3; 22.8]
Ambient ${NO}_{2}$	25.7 [17.7; 32.6]	25.4 [16.6; 33.1]	26.4 [19.0; 34.1]
Lung function parameters at two months of age			
FRC	99.1 [90.6; 109]	98.9 [90.2; 109]	98.7 [90.2; 109]
LCI	6.66 [6.06; 7.24]	6.64 [6.06; 7.23]	6.64 [6.03; 7.24]
TV	35.5 [30.8; 40.5]	35.5 [30.8; 40.5]	35.7 [30.8; 40.6]
RR	44.5 [38.4; 52.4]	45.6 [39.1; 53.4]	44.8 [38.8; 52.6]
MV	1605 [1421; 1795]	1612 [1458; 1814]	1606 [1427; 1796]
tPTEF/tE	34.1 [28.5; 42.5]	34.4 [28.5; 42.3]	34.3 [28.4; 42.3]

For categorical variables, relative frequencies are indicated in parentheses. For continuous variables, the median is reported followed by the first and third quartiles in brackets. $N_{2}\text{MBV}$: nitrogen multiple-breath washout test; TBFVL: tidal breathing flow-volume loops analysis.

### Ethics

The SEPAGES cohort received approval from the Ethics Committee (CPP, n°13-CHUG-4) Sud-Est V and the National Commission on Informatics and Liberty (CNIL, n° 914138). The Gen3G cohort was approved by Le Comité d’éthique de la recherche du CIUSSS de l’Estrie (CHUS, n° 2010-198, 07-027-A1). The Glowing clinical trial was approved by The University of Arkansas for Medical Sciences Institutional Review Board and registered under the number NCT01131117 (https://clinicaltrials.gov/study/NCT01131117). In each cohort, participants provided written informed consent prior to inclusion in accordance with the Declaration of Helsinki.

### Air pollution exposure assessment

Women carried wearable devices to measure exposure to ${\text{NO}}_{2}$ (passive sampler from Passam AG, Männedorf, Switzerland) and ${\text{PM}}_{2.5}$ mass concentration (MicroPEM active air sampler, RTI International, Research Triangle Park, NC, USA) during one to three separate weeks during pregnancy. Pregnancy exposures were estimated by averaging the measured concentrations from the personal sensors carried by each woman.^4^ Additionally, the PM_2.5_ OP per air volume were measured using dithiothreitol (${\text{OP}}^{\text{DTT}}$) and ascorbic acid (${\text{OP}}^{\text{AA}}$) assays on PM filters as previously described.^3^^,^^15^

We checked whether our findings still hold when PAPE was estimated through ambient exposures. Outdoor daily prenatal ${\text{PM}}_{10}$, ${\text{PM}}_{2.5}$ and ${\text{NO}}_{2}$ exposure levels were assessed at participants’ residential address using the fine spatial resolution dispersion model SIRANE that simulates the transport of pollutants across the streets at the district scale. It is based on an urban geometry, modelled via a network of connected street segments, meteorological parameters, background concentration of pollutants advected into the model domain by the wind and the emissions within each street in the network. The dispersion of pollutants across the streets is modelled using a Gaussian plume model.^16^ Pregnancy ambient exposures were estimated by averaging daily outdoor exposure levels over the pregnancy.

### DNA methylation measurement

Placental tissue was collected by the midwives of the study using a standardised procedure. Samples of approximately 5 mm^3^ were obtained from the foetal side, a few centimetres from the insertion of the cord, and frozen at −80 °C. DNA concentrations were determined in duplicate using the Quant-IT kit (ThermoFisher, Asnières-sur-Seine, France). Samples with discordant results were verified in a second series of measurements. DNA quality and correspondence with sample characteristics were evaluated on a subset of the samples by 1) migrating a small amount of DNA on a TapeStation 4200 (Agilent, Les Ulis, France) to calculate the DNA Integrity Number, 2) assessing amplification efficiency through the simultaneous amplification of two microsatellites markers, and 3) confirming the sex of individuals using PCR-based verification. 0.5–1 μg of genomic DNA was bisulfite treated with the EpiTect® Fast 96 DNA Bisulfite Kit (Qiagen, Courtaboeuf, France) according to the manufacturer’s instructions. After the conversion, samples’ volumes were adjusted based on the starting amount of DNA to normalise the sample concentration. The samples were processed with the DNeasy Blood & Tissue Kit (Qiagen) using the DNeasy® Blood & Tissue Handbook according to the manufacturer’s instructions. Whole-genome DNAm was measured using the Infinium HumanMethylationEPIC BeadChip (EPIC). Raw intensities of fluorescent signals were extracted using the GenomeStudio® software. The DNAm level of each CpG was calculated as the ratio of the intensity of fluorescent signals of the methylated alleles over the sum of methylated and unmethylated alleles (beta-value). No sample had to be excluded due to low call rate (more than 5% of probes with detection p-value > 0.01). Probes with detection p-value > 0.01 in at least one sample were filtered out. Raw intensities in beta-values were normalised using the *interpolatedXY adjusted funnorm* method.^17^ We ensured that placental DNAm-based sex prediction matched to the annotated sex. All CpG sites at a distance ≤2 bp from SNPs with minor allele frequency >0.05 were removed using the R package *DMRcate* (version 3.4.1).^18^ Cross-hybridised probes identified in previous studies were filtered out using the R package *maxprobes* (version 0.0.2; https://github.com/markgene/maxprobes). To reduce the influence of outliers, within each probe, the 1% most extreme methylation values were winsorised (imputed to the percentiles 0.05% and 99.5%). For all subsequent data analyses, beta-values were transformed to M-values. CpG sites were annotated to their closest gene based on the annotation provided in the R/Bioconductor package *IlluminaHumanMethylationEPICanno.ilm10b4.hg19* (version 0.6.0).

### Lung function assessment

To investigate the physiological state of the lungs and airways, noninvasive techniques were used to measure six lung function parameters in two-month-old children. As described elsewhere,^3^^,^^4^ the functional residual capacity (FRC) and the lung clearance index (LCI) were measured using nitrogen multiple-breath washout test ($N_{2}\text{MBV}$); the tidal volume (TV), respiratory rate (RR), minute ventilation (MV), and time to reach peak tidal expiratory flow as a proportion of total expiratory time ratio (tPTEF/tE) were assessed using tidal breathing flow-volume loops analysis (TBFVL). The FRC measures the volume of air in the lung after expiration and is an indicator of the lung volume. The LCI measures ventilation inhomogeneity in the peripheral airways and is used as a predictor of early airway diseases in children.^19^ The TV is the amount of air entering the lungs during a normal breath, the RR is the number of breaths in a minute and the MV is the volume of air entering the lungs in a minute (such that MV = RR x TV). A decrease in the FRC, TV or tPTEF/tE and an increase in the LCI can be interpreted as a decreased lung function. An increase in the MV and RR may indicate a higher respiratory need, and is interpreted as a decreased lung function.^5^ The flow chart of exclusions is shown in Supplementary Fig. S1.

### Statistics

We used median and interquartile range (IQR) or frequency (%) for the summary statistics. The relationship among air pollutants and between lung function parameters was evaluated using Spearman rank correlation.

The association between each air pollutant and each lung function parameter was investigated separately using linear regression models adjusted for potential confounders selected a priori: child sex (male/female; extracted from child health record), maternal age (continuous), maternal parity (binary: nulliparous/one child or more), maternal pre-pregnancy body mass index (BMI; continuous), maternal smoking during pregnancy (smoker/non-smoker), and parental highest educational attainment (binary: <master level, ≥master level), child height at the exam (continuous), child passive smoking (binary: yes/no), parental antecedents of rhinitis (binary: yes/no), season of conception (January–March, April–June, July–September, October–December) and for the European Ecological Deprivation Index (EDI, as a measure of neighbourhood socioeconomic status; continuous).

For each pair of air pollutant and lung function parameter separately, two levels of mediation analyses were conducted: CpG-wise and regional mediation analyses using HDMAX2^11^ (Fig. 1). This approach, tailored for high-dimensional mediation analysis, first performs an Epigenome-wide Association Studies (EWAS) between the exposure and the mediator and an EWAS between exposure and the outcome adjusted for the mediator. Each EWAS relies on a regression model (models M1 and M2 respectively) computed using the lfmm method (version 1.1).^20^ HDMAX2 then combines paired p-values from the two EWAS to calculate FDR-corrected p-values for each CpG separately and identify the most credible candidate mediators. Causal indirect and direct effects and nominal p-values for the selected CpG sites are then computed using the R package mediation (version 4.5.1).^21^ It has been observed that setting an FDR < 0.05 can be overly conservative in high-dimensional mediation analyses, we thus rather selected, as significant mediators, the CpGs with an FDR < 0.2, as previously suggested.^11^ AMRs are detected via the comb-p method, which aggregates adjacent CpG nominal paired p-values using sliding windows and Stouffer-Liptak-Kechris correction.^11^^,^^22^ HDMAX2 additionally estimates the part explained by all mediators altogether (called the overall IE; OIE) by combining them in a regression model. Both models M1 and M2 were adjusted for putative confounding factors. The model M1 was adjusted for: child sex (male/female), maternal age (continuous), maternal parity (binary: nulliparous/one child or more), maternal pre-pregnancy body mass index (BMI; continuous), maternal smoking during pregnancy (smoker/non-smoker), and parental highest educational attainment (binary: <master level, ≥master level). The model M2 was adjusted for all adjustment factors included in model M1, plus: child height at the exam (continuous), child passive smoking (binary: yes/no), parental antecedents of rhinitis (binary: yes/no), season of conception (January–March, April–June, July–September, October–December) and for the European Ecological Deprivation Index (EDI, continuous). Further confounding in DNAm data induced by technical effects and differences in cell-type composition of placenta samples were accounted for by adjusting both mediation models for six latent factors computed using *lfmm2*.^11^^,^^20^ To reduce the dimensionality of the data and the risk of false positive detections, we identified reliable CpGs based on technical replicates (see below). CpG-wise mediation analyses were restricted to the CpGs for which the inter-individual variance accounted for more than 50% of the total variance (42% of the CpGs were excluded; 437,964 remaining CpGs). AMRs were selected using the comb-p procedure applied to max2 p-values for the indirect effect, with default parameters (maximum distance to combine adjacent CpGs: 1000 base pairs; significance threshold for initiating a region: 0.01).^23^ Selected AMRs were required to have an FDR < 0.05 and to contain at least three probes, including at least one reliable probe (inter-individual variance >50% of the total variance). For each selected AMR, the causal indirect effect was subsequently re-estimated using the mean methylation level and tested for significance (nominal p-value < 0.05; bootstrap test). FDRs were computed using the Benjamini-Hochberg method. Missing values in clinical covariates were imputed using simple imputation and the R package Amelia II (version 1.8.3),^24^ based on information from all included clinical covariates, exposures and outcomes. All analyses were performed using the R software (version 4.3.3).

### Selection of informative probes

As presented elsewhere,^8^ we performed a variance component analysis based on samples with at least one technical replicate (fourteen samples for which two to five technical replicates were available). Variance components were estimated via a linear mixed model adjusted for the batch effect, as follows: M-value = (1| individual) + experiment. This allowed us to decompose the total variance into inter-individual, intra-individual (i.e.: measurement error) and batch variance as such: total variance = inter-individual variance + batch variance + error variance; and to evaluate whether the variance in M-values is predominantly influenced by differences between individuals or by technical effects. The measurements of a probe were considered reliable when most of the variance could be attributed to inter-individual variability rather than intra-individual and batch variability (inter-individual variance >50% of total variance). The reliable probes are provided in the Supplementary Material (Supplementary Table S11).

### Expected number of false positive detections

The expected number of false positive detections for a given FDR threshold θ is computed a posteriori, based on the number of detections (*d*) that fell below the threshold. As the FDR controls the proportion of false positive detections, the expected number of false positive detections is estimated to be, in average, less than: d∗ θ.

### Consistency analyses

We evaluated whether our findings (CpGs and AMRs) were consistent across air pollutants (personal and ambient) and lung function parameters. A mediator was considered consistent for another pair of air pollutant and lung function parameter if the IE was significant (nominal p-value < 0.05) and had the same direction of the effect.

### DNAm mediators and gene expression

Expression-associated quantitative trait methylation eQTM analysis identifies CpG sites at which DNAm is associated with gene expression. To evaluate whether our significant mediating CpGs and AMRs were likely to contribute to the regulation of gene expression, we queried them in a database of (proximal) cis-eQTMs (±0.5 Mb). This database was derived from matched placental DNAm and RNA-seq gene expression datasets from the mother-child cohorts Gen3G and GLOWING (see below). These cis-eQTMs loci were highlighted as they may be more likely to contribute to the regulation of (proximal) gene expression.^25^

### Expression quantitative trait methylation

An eQTM analysis was performed in the Gen3G (Canada; n = 176) and GLOWING (USA; n = 150) cohorts.^26^^,^^27^ Gene expression levels were assessed via mRNA-sequencing in matched biopsies, collected from the same regions of the placenta than the samples in which DNAm levels were measured. Reads were aligned against the human genome hg38. Genes were annotated using GENCODE v28 and v30 for GLOWING and Gen3G respectively. DNAm was measured using the Infinium HumanMethylationEPIC *BeadChip*. Cis-eQTMs were identified using the Torch-eCpG tool, considering CpGs located within 0.5 Mb (1 Mb window) up- and downstream of the TSS of each gene.^28^ Analyses were restricted to children at term and without pregnancy complications. For each pair of CpG and gene, the association between DNAm levels and each gene expression levels were tested using multivariate linear regression models, adjusted for child sex, child ancestry, gestational age at delivery, six reference cell type proportions estimated *in-silico* (R package *planet;* version 1.16.0),^29^ DNAm contamination score and mRNA-seq PCs. Results from the cohorts were combined using fixed-effects inverse variance weighted meta-analysis. In total, 9,293,948 CpG-gene pairs were considered. The pairs present in only one cohort were excluded (46.2%; leaving 4,783,66 CpG-gene pairs to be tested). The cis-eQTMs were defined as the CpGs significantly associated with the expression level of one gene located within a window of ±0.5 Mb (p-value < 0.01).

### Link with imprinted genes

Placental imprinted genes are considered important regulators of foetal growth and their expression has been associated with maternal exposure to air pollution.^30^ We sought whether the CpGs and AMRs mediating the relationship between PAPE and lung function parameters overlapped with known imprinted genes or whether their DNAm levels were associated with the gene expression level of known proximal imprinted genes in the cis-eQTM database described above. We made use of the list of imprinted genes identified in humans which have been recorded in the www.geneimprint.com and https://corpapp.otago.ac.nz/gene-catalogue databases, as previously described.^31^

### Gene set enrichment analyses

To get further insight into the biological pathways particularly affected by the significantly mediating CpGs and AMRs, we searched for enriched pathways within the KEGG 2021 and Reactome 2022 databases, for enriched Gene Ontologies (GO terms for biological processes, 2023) and for enriched health outcomes and phenotypes within the database of Genotypes and Phenotypes—dbGAP. All the databases are part of the R package *enrichR* (version 3.4) and were downloaded at https://maayanlab.cloud/Enrichr/#libraries. Only pathways and entries including more than 20 genes were considered. Gene set enrichment was tested using the method implemented in the R package *missMethyl* (version 1.42.0), which corrects for probe overrepresentation bias.^32^

### Robustness and sensitivity analyses

We evaluated whether our hits were robust to extreme values by re-computing the models after winsorising 1% extreme values in the exposures, DNAm and the lung function parameters. Moreover, as ${\text{NO}}_{2}$ is a strong oxidant, we evaluated whether the hits associated with ${\text{PM}}_{2.5}$ OP levels were robust when statistical models were further adjusted for personal ${\text{NO}}_{2}$ exposure. A DNAm mediator was considered robust if: (1) the causal IE remained statistically significant (nominal p-value < 0.05), (2) the direction of the causal IE did not change (3) and if the percent change in effect size in the sensitivity analysis compared to the main analysis was below 20%.

Moreover, causal mediation analysis relies on the untestable assumption that there is no unmeasured pre-treatment confounder between the mediators and the outcomes (named the sequential ignorability assumption). Sensitivity analyses were conducted to evaluate empirically whether our results were sensitive to the violation of this assumption. For so doing, we applied the correlated residuals method implemented in the function *medsens* from the R package *mediation* (version 4.5.1).^21^^,^^33^ This method relies on the idea that the correlation ρ between error terms of the two mediation models M1 and M2 (defined above) can be used as a measure of the degree of unobserved pre-treatment confounding (and therefore of the degree of violation of the sequential ignorability). In particular, if there is an omitted pre-treatment confounder, the correlation (ρ) between the two errors terms will be different from zero. To evaluate how robust the results of the mediation are to the violation of the sequential ignorability assumption, the approach examines how the indirect effect (IE) changes for varying values of the correlation ρ between −1 and 1. Results that become statistically insignificant, or even change signs, with a small degree of violation of the assumption (values of ρ values close to 0) are considered to be sensitive and unreliable.

Supplementary figures and tables are labelled as Supplementary Figs. S1–S7 and Supplementary Tables S1–S10, respectively, and are provided in the Supplementary Material.

### Role of funders

Funders had no role in study design, data collection, data analyses, interpretation nor writing of the manuscript.

## Results

### Study population

In average, gestational duration was 40 weeks and two-month-old infants’ weight was 4.8 kg. In 70% of couples, at least one of the parents had at least a Master’s degree (or equivalent). The average pregnancy exposures to ${\text{PM}}_{2.5}$ ${\text{OP}}^{\text{DTT}}$ and ${\text{OP}}^{\text{AA}}$ were 1.50 and 1.56 $\text{nmol}/\left(\min*m^{3}\right)$ respectively (Table 1 and Supplementary Fig. S2). Average ambient and personal ${\text{PM}}_{2.5}$ exposure levels were equivalent (13.5 $\mu g/m^{3}$ and 13.2 $\mu g/m^{3}$ respectively) but they were lowly correlated (rho = 0.18; Supplementary Fig. S3). The average personal ${\text{NO}}_{2}$ exposure (19.2 $\mu g/m^{3}$) was lower than the ambient one (25.7 $\mu g/m^{3}$) and they showed modest Spearman correlation (rho = 0.54). The demographic and exposure characteristics of the subsets having both DNAm and lung function measurements (N = 297 for $N_{2}\text{MBV}$ and N = 267 for TBFVL) were not significantly different from the characteristics of the whole cohort (Table 1 and Supplementary Table S1). Lung function parameters were not strongly correlated. The highest (Spearman) anti-correlation was observed between TV and RR (rho = −0.62) and the highest correlation between TV and LCI (rho = 0.61) (Supplementary Fig. S4). Distributions of cell type proportions across air pollutant levels are shown in Supplementary Fig. S5.

### Associations between air pollutants and lung function parameters

We observed a negative (adverse) trend of association between the FRC and all the exposures except ${\text{PM}}_{2.5}$ ${\text{OP}}^{\text{AA}}$. More specifically, we found negative trends of association between FRC and both personal ${\text{PM}}_{2.5}$ concentration and ${\text{OP}}^{\text{DTT}}$ exposure (β = −0.21, nominal p-value < 0.13 and β = −1.91, nominal p-value < 0.14 respectively; Wald test), which is in line with previous results from the SEPAGES cohort obtained on a slightly larger sample size.^3^^,^^4^ Additionally, significant negative associations were found between FRC and ambient ${\text{PM}}_{2.5}$ and ${\text{PM}}_{10}$ exposure (β = −0.60, nominal p-value < 0.04 and β = −0.60, nominal p-value < 0.03 respectively; Wald test). For the other parameters, association results were less consistent across air pollutants, though a significant (nominal p-value < 0.05; Wald test) negative association was found between personal ${\text{NO}}_{2}$ exposure and MV (Supplementary Fig. S6 and Supplementary Table S2).

### Mediation analyses

**Fig. 2:**
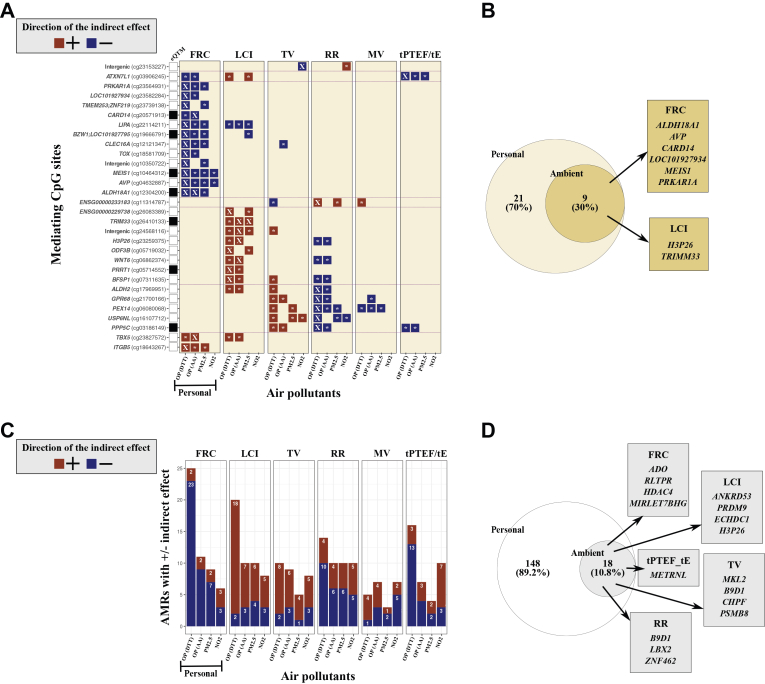
**A.** CpGs significantly mediating the relationship between PAPE and child lung function parameters and consistency of the mediators across personal exposures and lung function parameters. A black box in the left-hand column indicates the CpG is an eQTM. A white cross indicates the site was detected by HDMAX2 (FDR < 0.2 after correction for multiple tests) and has a significant indirect effect (IE; nominal p-value < 0.05; bootstrap test) for the corresponding air pollutant, a white star indicates the IE of the site was found nominally significant (nominal p-value < 0.05 but FDR > 0.2). The colour indicates the direction of the IE (blue: negative IE; dark red: positive IE). **B.** Overlap between mediating CpGs detected with personal exposures and mediating CpGs replicated with ambient exposures. A CpG was considered replicated if its causal IE had nominal p-value < 0.05 (bootstrap test). Known genes annotated to a CpG consistent between personal and ambient exposures are displayed. **C.** Number of significant AMRs with a negative/positive (blue/red) indirect effect. **D.** Overlap between AMRs detected with personal exposures and AMRs replicated with ambient exposures. An AMR was considered replicated if its causal IE had nominal p-value < 0.05 (bootstrap test). Known genes annotated to an AMR consistent between personal and ambient exposures are displayed. For lung function parameters LCI and FRC, sample numbers were: N = 237 for ${\text{PM}}_{2.5}$ OP assays, N = 249 for personal ${\text{PM}}_{2.5}$ and N = 297 for personal ${\text{NO}}_{2}$ and N = 289 for ambient ${\text{PM}}_{2.5}$ and ambient ${\text{NO}}_{2}$ and N = 295 for ambient ${\text{PM}}_{10}$. For lung function parameters TV, RR, MV and tPTEF/tE, sample numbers were: N = 255 for ${\text{PM}}_{2.5}$ OP assays, N = 264 for personal ${\text{PM}}_{2.5}$, N = 267 for personal ${\text{NO}}_{2}$, N = 261 for ambient ${\text{PM}}_{2.5}$ and ambient ${\text{NO}}_{2}$ and N = 265 for ambient ${\text{PM}}_{10}$.

In total, we found 30 distinct CpGs significantly mediating (FDR < 0.2) the relationship between at least one air pollutant and one lung function parameter, including fourteen CpGs associated with the FRC, eight with the LCI, six with the RR, one with the TV and one with tPTEF/tE (Fig. 2A and Supplementary Table S3). Most of these sites were detected in association with personal ${\text{PM}}_{2.5}$ ${\text{OP}}^{\text{DTT}}$ and ${\text{OP}}^{\text{AA}}$ exposure (23/30 and 7/30 respectively). Nine of the 30 mediating CpGs (30%) were consistent between personal pollutants (or OP) and ambient air pollutants (six CpGs for FRC, three for LCI; Fig. 2B and Supplementary Table S4). Additionally, seven (out of 30; 23.3%) of the significant mediating CpGs were found to be significantly associated with the expression level of proximal genes (three with the expression of their cognate gene) in the placental eQTMs database (Table 2). There was no detection below the FDR threshold of 0.05 and 0.1. But, for 30 detections below the FDR threshold of 0.2, we expect on average less than six false positive calls. We further detected 166 non-overlapping AMRs significantly mediating the relationship between at least one personal exposure and one outcome (Fig. 2C and Supplementary Table S5). In total, 18 AMRs (10.8%) were found consistent with at least one ambient exposure (Fig. 2D and Supplementary Table S6). Moreover, 73 out of the 166 regions (44%) encompassed at least one eQTM, including 19 regions for which the eQTM was associated with the expression level of its cognate gene (Supplementary Table S7).

**Table 2:** The seven significant mediating CpGs found to be significantly associated with gene expression (Wald test; nominal p-value < 0.01) in the placental eQTM database.

CpG	Outcome	Exposure	Indirect effect estimate	Gene name of the CpG site	Name of the gene associated with DNAm	Effect size of the eQTM	eQTM nominal p-value	Relevant related biological functions
cg12304200	FRC	${\text{OP}}^{\text{AA}}$	−0.99	*ALDH18A1*	*PDLIM1*	3.37	0.001	Inflammatory response
cg20571913	FRC	${\text{OP}}^{\text{AA}}$	−0.69	*CARD14*	*SLC26A11*	4.07	0.003	Coding for a protein essential for cell homoeostasis
cg10464312	FRC	${\text{OP}}^{\text{DTT}}$	−0.97	*MEIS1*	*MEIS1*	−2.53	0.007	Involved in lung development
cg12304200	FRC	${\text{OP}}^{\text{DTT}}$	−1.37	*ALDH18A1*	*PDLIM1*	3.37	0.001	Inflammatory response
cg19666791	FRC	${\text{OP}}^{\text{DTT}}$	−0.85	*BZW1*	*AOX1*	−4.83	0.008	Regulation of ROS
cg26410133	LCI	${\text{OP}}^{\text{AA}}$	0.04	*TRIM33*	*TRIM33*	−2.99	0.010	Protection against oxidative stress
cg05714552	LCI	${\text{OP}}^{\text{DTT}}$	0.06	*PRRT1*	*HSPA1B*	−4.41	0.003	Cellular stress response
cg26410133	LCI	Personal ${\text{PM}}_{2.5}$	0.007	*TRIM33*	*TRIM33*	−2.99	0.010	Protection against oxidative stress
cg03186149	RR	${\text{OP}}^{\text{DTT}}$	−0.64	*PPP5C*	*PPP5C*	−3.95	3.2E-08	Regulation of cellular stress

FRC: Functional Residual Capacity; LCI: Lung Clearance Index; RR: Respiratory Rate; OP: Oxidative Potential of the ${\text{PM}}_{2.5}$; eQTM: expression quantitative trait methylation; ROS: Reactive Oxygen Species.

Overall, except for TV and RR which shared 19 AMRs, the mediating CpGs and AMRs identified in our analysis appear to be specific to each lung function parameter (Fig. 2A and Supplementary Fig. S7A and B). For this reason, we will examine the results in more details for each lung function parameter separately.

### Nitrogen multiple-breath washout test ($N_{2}MBV$) parameters

#### Functional residual capacity (FRC)

We identified 14 distinct CpGs significantly mediating the relationship between ${\text{PM}}_{2.5}$ OP and FRC. These included 11 sites explaining a negative (adverse) effect and one site explaining a positive (favourable) effect of ${\text{OP}}^{\text{DTT}}$ on FRC, and 3 sites mediating the effect of ${\text{OP}}^{\text{AA}}$ on FRC (one shared with ${\text{OP}}^{\text{DTT}}$) (Supplementary Table S3). No significant mediator was detected for the other personal air pollutants. Nonetheless, 3 of the 14 significant mediators were consistent across personal ${\text{PM}}_{2.5}$ OP and mass concentration and ambient ${\text{PM}}_{2.5}$ mass concentration, though the magnitude of the IE was markedly higher for OP indicators (Fig. 2A and Supplementary Table S3). These included an eQTM (cg10464312; Table 2) mapping and associated to the expression of the gene *MEIS1*, a CpG (cg046328*8*7) located in the TSS of the gene *AVP* and one site (cg12304200) annotated to the gene *ALDH18A1*.

The regional analyses identified 39 non-overlapping AMRs mediating the relationship between FRC and at least one personal exposure (25 regions mediating the effect of ${\text{PM}}_{2.5}$ ${\text{OP}}^{\text{DTT}}$). Five (out of the 25) OP-related AMRs encompassed eQTMs associated with the expression of their cognate gene: *MEIS1* (also detected in the CpG-wise analysis), *AKIP1*, *ACTR3C, TAS1R3, GPX3* and *LZTFL1* (all with an adverse IE). Additionally, we found AMRs (with adverse IE) annotated to genes playing important roles in lung development: *DPPA2* and *COL6A1*^34^^,^^35^ (Supplementary Tables S5 and S7). In line with the CpG-wise analysis, the majority of the AMRs mediated an adverse effect of PAPE on the FRC so that the overall mediating effect of DNAm between OP of the ${PM}_{2.5}$ and ${PM}_{2.5}$ mass exposures and the FRC was in the negative direction (Supplementary Table S8). Nevertheless, we found two significant CpGs with a positive (protective) IE, annotated to the genes *ITGB5* and *TBX5*.

#### Lung clearance index (LCI)

We detected eight CpGs and 38 AMRs significantly mediating the relationship between PAPE (especially for ${\text{PM}}_{2.5}$ OP) and the LCI. These included 4 sites explaining an adverse (positive) effect of ${\text{OP}}^{\text{DTT}}$ and 4 sites mediating an adverse effect of ${\text{OP}}^{\text{AA}}$ on LCI (Supplementary Table S3). Two (out of the eight) CpGs were also eQTMs, associated with the expression of the genes *TRIM33* and *HSPA1B1* (Table 2). Moreover, 17 (out of the 38) AMRs overlapped with an eQTM, including regions annotated and associated with the expression of the genes *CARNS1* and *DIP2C* (adverse IE) (Supplementary Tables S5 and S7). All of the mediating CpGs and a majority of the AMRs accounted for an adverse (positive) effect of PAPE on the LCI, though the (nonsignificant) total effect was in the negative direction (Supplementary Table S8).

### Tidal breathing parameters

Overall, we identified less mediating CpGs for TBFVL parameters than for $N_{2}\text{MBV}$ parameters. Furthermore, for all TBFVL parameters except TV, we observed nearly as much AMRs with adverse than with favourable IE (Fig. 2A and C).

#### Tidal volume (TV)

One CpG (intergenic) and 28 AMRs were found to significantly mediate the relationship between PAPE and TV. Overall, 13 of the AMRs overlapped with an eQTM, including one region (with an adverse IE) both annotated and associated to the expression of the gene *ARID5A.* A majority of the AMRs accounted for a positive (i.e., protective) effect of PAPE on the TV, though the total effects were non-significant (Supplementary Fig. S6). Consistently, the overall mediating effect of DNAm between ${\text{PM}}_{2.5}$ OP exposure and the TV was in the positive direction (Supplementary Table S8).

#### Respiratory rate (RR)

We identified six CpGs which significantly mediated the relationship between PAPE and the *RR*, including 5 sites explaining a negative effect and one site explaining a positive effect of ${\text{OP}}^{\text{DTT}}$. These results included an eQTM, annotated to the gene *PPP5C* (decreased RR; Table 2). Moreover, 35 AMRs were detected, 21 of which encompassed at least one eQTMs, including three mediator regions annotated and associated to the expression of the genes: *DIP2C* (negative IE), *NXPH4* (decreased RR) and *CROT* (increased RR) (Supplementary Table S7). The overall IE of DNAm in the relationship between all air pollutants and ${\text{PM}}_{2.5}$ OP and the RR was negative (Supplementary Table S8).

#### Minute ventilation (MV)

We found no significant CpG site but 19 AMRs significantly mediated the relationship between PAPE and the MV including seven regions encompassing at least one eQTM, and three which were both annotated and associated to the expression of the genes *RGS14*, *DIP2C* (protective IE) and *CCHCR1* (adverse IE) (Supplementary Table S7). The overall mediating effect of DNAm between ${\text{PM}}_{2.5}$ OP and MV was positive (adverse) whereas it was negative (favourable) between personal ${\text{PM}}_{2.5}$ concentration and ${NO}_{2}$ and MV (Supplementary Table S8).

#### Ratio tPTEF/tE

One CpG (gene *ATXN7L1*, adverse IE) and 33 AMRs were found to significantly mediate the relationship between PAPE and tPTEF/tE. In total, 14 AMRs overlapped with an eQTM, including regions both annotated and associated to the expression of the genes *N4BP2L1* (adverse IE), *METRNL, MKRN3*, *KDM6B, ARL4D,* and *DDX60* (favourable IE; Supplementary Table S7). The overall mediating effects were not consistent across exposures (negative for personal ${\text{PM}}_{2.5}$ ${\text{OP}}^{\text{DTT}}$ and mass concentration but positive for ${\text{OP}}^{\text{AA}}$ and personal ${\text{NO}}_{2}$; Supplementary Table S8).

### Overlap between DNAm mediators and imprinted genes

In total, we found four AMRs annotated to an imprinted gene. One AMR was annotated to the maternally imprinted gene *ALDH1L1* (in the liver). The three others were annotated to the paternally (placental) imprinted genes *DNMT1, GLIS3* and *MKRN3*. Moreover, DNAm levels of the AMR annotated to the gene *MKRN3* were also significantly associated with gene expression (eQTM). Interestingly, the direction of the IE for these three AMRs indicated a protective (or a positive adaptation) effect of PAPE-driven DNAm changes on lung function (Supplementary Table S5).

### Gene set enrichment analyses

The set of CpG and AMRs mediating the effect of personal ${\text{NO}}_{2}$ exposure on the MV was found significantly enriched (FDR < 0.05) for genes implicated in the KEGG pathways “estrogen signaling”, and “lipid and atherosclerosis”, as well as pathways involved in the response to infection (legionellosis, measles, prion disease). In addition, the set of ${\text{OP}}^{\text{AA}}$-RR mediators was found significantly enriched in the KEGG pathway “arachidonic acid metabolism” (Supplementary Table S9). In the dbGap database, ${\text{OP}}^{\text{AA}}$-LCI mediators were found enriched for genes related to amytrophic lateral sclerosis and the sets of ${\text{OP}}^{\text{AA}}$-MV, ${\text{OP}}^{\text{AA}}$-RR and ${\text{NO}}_{2}$-MV mediators were significantly enriched for genes associated with gamma-glutamyltransferase and alkaline phosphatase (Supplementary Table S10). No significant enrichment was found for Reactome pathways nor GO terms.

### Robustness of the results

Most of the detected mediators were robust to winsorisation of extreme values. Indeed, 24 CpGs (out of 30) remained detected by HDMAX2 (FDR-corrected p-value < 0.2). Moreover, all the CpGs except one intergenic site (with a 23% change) and 143 AMRs (out of 166) complied with our criteria for robustness. Additionally, all the mediator CpGs and 99 out of the 100 AMRs associated with ${\text{PM}}_{2.5}$ OP models were robust to adjustment for personal ${\text{NO}}_{2}$ exposure (Supplementary Tables S3 and S5).

The absolute values for the sensitivity measure ρ were comprised between 0.1 and 0.3, with most CpGs (respectively most AMRs) having values between 0.2 and 0.3 (0.15 and 0.3 respectively) (Supplementary Tables S3 and S5). A higher absolute value of ρ indicates greater robustness of the results. A value of 0.3 has previously been interpreted as a modest level of sensitivity to the departure from the sequential ignorability assumption.^36^ However, to our knowledge, there are no cut-off nor reference values indicating a questionable degree of susceptibility to mediator-outcome confounding in other DNAm mediation studies at the time.

## Discussion

Leveraging data from the French SEPAGES cohort, our epigenome-wide screening identified placental DNAm changes that may mediate the relationship between prenatal exposure to air pollution and six lung function parameters measured in newborns. We found that both site-specific and regional placental DNAm changes significantly mediated the effect of personal PAPE on lung function. One third of the mediating CpGs and 11% of the AMRs were also detected with ambient exposures. Strikingly, we detected more and larger mediating effects for prenatal personal exposure to ${\text{PM}}_{2.5}$ OP than for the other exposures. In particular, most DNAm modifications were found to mediate the relationship between ${\text{PM}}_{2.5}$ OP and the FRC and LCI. We observed that prenatal exposure to ${\text{PM}}_{2.5}$ OP mainly had adverse effects on the FRC and LCI through changes in DNAm, although we also detected a few DNA modifications that might mediate positive adaptive effects on lung function. Interestingly, several AMRs located within imprinted genes were identified as mediators of protective or enhancing effects on infant lung function. Moreover, the total effect of personal PAPE on lung function parameters was nonsignificant. As extensively discussed in previous works, significant mediation can occur in absence of a significant total effect and this may suggest that the mainly deleterious DNAm-mediated effects might be counterbalanced by other mechanisms. A complementary expression quantitative trait methylation (eQTM) analysis suggested that nearly one quarter of the mediating CpGs and one half of the AMRs could have a functional impact by regulating gene expression in the placenta.

The lung function of infants is partly determined by in-utero environmental exposures and proper foetal airway development.^1^ In keeping with this, our analysis revealed several PAPE-mediating DNAm changes related to genes known to be involved in lung development and in lung function, including the genes *ITGB5* (associated to airway responsiveness) and *TBX5* (involved in the regulation of lung growth and branching).^37^^,^^38^ One of our most salient discoveries was that an adverse effect of pregnancy ${\text{PM}}_{2.5}$ OP exposure on the FRC was significantly mediated by DNAm modifications at eQTM CpG sites located in the body of the gene *MEIS1*. The *MEIS1* gene is a homeobox gene and a transcription factor that plays important and broad-ranging roles in normal development.^39^ Interestingly, a previous study has shown that mice mutant for this gene displayed hypoplastic lungs.^40^^,^^41^ Decreased expression of *Meis1* in rat foetal lungs and diaphragms was further associated with a reduction in mesenchymal cell proliferation leading to malformed pleuroperitoneal folds and disrupted airway branching.^42^ In addition, the negative relationship between ${\text{PM}}_{2.5}$ OP and FRC was further explained by DNAm changes annotated to the genes *DPPA2* and *COL6A1*, which are involved in lung development.^34^^,^^35^ These findings suggest that the acknowledged adverse effect of PAPE on newborn FRC could be partly explained by DNAm alterations in genes implicated in lung development. Furthermore, our findings highlighted that an adverse effect of pregnancy ${\text{PM}}_{2.5}$ OP on the LCI could be mediated by changes in the DNAm levels of loci associated with the expression of genes associated with pulmonary diseases such as *TRIM33* and *DIP2C.* These observations are in line with the interpretation of the LCI as an indicator of abnormal lung function in children.

PAPE is deemed to affect the foetal lung function by influencing the immune system and inducing systemic inflammation.^1^ Accordingly, our findings highlighted that the effect of ${\text{PM}}_{2.5}$ OP on newborn lung function was mediated by placental epigenetic changes related to genes involved in inflammation and in the immune response. This included DNAm-mediated changes in the gene *TAS1R3*, a taste receptor that regulates human respiratory innate immunity, at the gene *ARID5A,* involved in the regulation of the inflammatory response and auto-immunity processes; and in the gene *KDM6B* involved in inflammatory response. In addition, we observed that the effect of ${\text{PM}}_{2.5}$ OP on most of the lung function parameters was also significantly mediated by placental epigenetic changes related to genes implicated in cellular response to stress and notably in oxidative stress, including the genes *GPX3, HSPA1B1, CARNS1, PPP5C* and *RGS14*. Oxidative stress plays an important role in placental homoeostasis, a critical biological process enabling the cells to buffer against the effects of environmental variations.^43^ In line with this, we found multiple DNAm-mediated changes in genes involved in homoeostasis, including the gene *AVP* (adverse effect) which encodes a neuropeptide likely involved in maintaining respiratory homoeostasis.^44^ In addition, the DNAm mediators for the RR and the MV were significantly enriched for genes associated with the production of gamma-glutamyltransferase and alkaline phosphatase, two enzymes related to the homoeostasis of oxidative stress.

Environmentally-induced placental epigenetic changes may not always be deleterious. They may also act as a mechanism for resilience by which the placenta adapts and triggers buffering responses that allow the foetus to achieve positive developmental outcomes.^6^^,^^9^ As mentioned above, the PAPE-driven placental DNA modifications implicated in inflammation and stress response were in certain cases positively adaptative (associated with increased lung function at two months). Interestingly, our findings highlighted four AMRs at known parentally imprinted genes (*ALDH1L1, DNMT1, GLIS3* and *MKRN3*) which all mediated a protective or enhancing effect on infants’ lung function. These results may reflect one of the putative roles of imprinted genes as potential key modulators of developmental plasticity and foetal adaptation to environmental cues.^45^ Furthermore, placental epigenetic changes can have enhancing effects on specific features of the lung function but deleterious effects on other features. For example, DNAm changes in the gene *DIP2C* mediated an adverse (increasing) effect of ${\text{PM}}_{2.5}$ OP on the LCI but also mediated a decrease of the MV. This gene has been associated with lung cancer and pulmonary function.^46^ More generally, placental epigenetic adaptations affecting newborn lung function could have protective or deleterious effects on the development of other foetal tissues, such as the brain or the cardiovascular system.^6^ Additional mediation analyses investigating other health outcomes in the offspring could help elucidate the overall impact of placental DNAm alterations on infants’ development and health.

Although prenatal air pollution has been extensively associated with impaired respiratory health in children, we detected few statistically significant total effects in our analyses of the association between PAPE and lung function parameters. Our analyses might have been underpowered by the modest sample size (N = 267–297). As previously shown, the test for the total effect can sometimes be less powerful than the test for an IE and, even in the absence of a significant total effect, investigating mediating epigenetic mechanisms can be warranted and provide insightful evidence of the intermediate regulatory events linking an environmental exposure to a health outcome.^10^^,^^12^^,^^13^ Accordingly, our analyses were able to identify significant mediating effects through placental DNAm related to relevant biological pathways, with a potential effect on gene expression. Nonsignificant total effects could also suggest other antagonistic mechanisms at work–either epigenetic (e.g., through histone modifications, chromatin remodelling and miRNA dysregulation) or not and involving the placenta or not–which would compensate for the effect mediated by placental DNAm changes.

This study has several important strengths. First, pregnancy air pollution exposure was assessed via personal active and calibrated captors that account for personal mothers’ exposure and using computational models that estimate ambient outdoor exposures. Secondly, we were able to assess and investigate the effect of the oxidative potential of the ${\text{PM}}_{2.5}$, which is expected to be a better indicator and predictor of their toxic effect on health.^3^ This redox property of the PM integrates different physicochemical characteristics of the PM such as its size distribution, solubility, composition and surface-mediated reactivity.^47^ This may explain why our analyses detected more and stronger mediation effects in association with the exposure to the ${\text{PM}}_{2.5}$ OP than with any other exposure. Moreover, ${\text{OP}}^{\text{DTT}}$ and ${\text{OP}}^{\text{AA}}$ are complementary measurements of ${\text{PM}}_{2.5}$ OP because they are representative of different lung antioxidant categories and are not sensitive to the exact same compounds of the ${\text{PM}}_{2.5}$. ${\text{OP}}^{\text{DTT}}$, as a thiol component, is reactive to a broad range of air pollutants within PM whereas ${\text{OP}}^{\text{AA}}$ is more specific especially to components from anthropogenic sources as biomass burning and traffic. Nonetheless, we obtained fairly consistent results between the two assays. We noted fewer statistically significant DNAm mediators in relation to personal ${\text{NO}}_{2}$ exposure than with exposure to ${\text{PM}}_{2.5}$ mass concentration and OP. This might be because ${\text{NO}}_{2}$ levels are a proxy for traffic-related air pollution and are more spatially heterogeneous than PM, whereas personal ${\text{PM}}_{2.5}$ exposure may better reflect both indoor and outdoor personal air pollution exposure. However, personal exposures cannot be recorded during the entire pregnancy. Two to three periods of one week during the pregnancy had to be sampled and pregnancy exposure was estimated by averaging the measurements obtained during each sampled week. Additionally, in contrast to most other studies that considered children older than five years, lung function parameters were measured very early in children (at two months). They may thus be less confounded by postnatal exposures or behaviours, allowing us to better evaluate the impact of PAPE on foetal lung development than measurements recorded later in children’s life. Nonetheless, we cannot rule out residual confounding factors due to the observational design of our study and the risk of false positive detections due to the multiple tests. For these reasons, our results need to be replicated in larger independent datasets from other geographical backgrounds and patterns of air pollution exposure. Moreover, there are known sex differences in both foetal lung function development and PAPE-driven placental epigenetic changes, as well as potential windows of increased susceptibility to PAPE.^8^ Due to the modest sample size and to the sampling method for personal exposures, we considered our data were not suited to perform robust enough sex-stratified and trimester-specific analyses. Future mediation analyses in larger cohorts would be of great interest to identify potential sex-specific mediators and critical windows of exposure susceptibility. Further analyses integrating DNAm with other epigenetic marks, such as miRNA and histone marks, would also prove valuable for gaining a more complete understanding of the role of placental epigenetic modifications in the relationship between prenatal PAPE exposure and infant lung function.

In conclusion, our results suggest that pregnancy exposure to air pollution, particularly ${PM}_{2.5}$ OP, may affect newborn lung function through multiple placental epigenetic changes involved in lung development, homoeostasis and in the activation of stress response mechanisms, including inflammation and oxidative stress. The results of the expression analyses supported a functional role for many mediators. We found that most of the changes in placental DNAm mediated the adverse effect of PAPE exposure on lung function. Nonetheless, a few PAPE-driven DNAm modifications, notably those located in known imprinted genes, could be positively adaptive and contribute to increased lung function in newborns.

## Contributors

LB and JL designed the current study and wrote the manuscript. VS and OF provided methodological support. JT supervised the measurement of DNAm. AB and VS assessed and verified lung function data. SG computed ambient exposures. AB curated the database. GU funded and provided personal measurements of oxidative potential. AM and CT curated the OP database. SAL, FW, KC, TME and MFH developed the external placental eQTM database. SLC, CP, SB and RS are the coordinators of the SEPAGES cohort. LB and JL accessed and verified all the underlying data. LB performed the statistical analyses. All authors critically reviewed and approved the final version of the manuscript.

## Data sharing statement

Complete results of the mediation analysis for each pair of air pollutant and lung function parameter are available on Zenodo at https://doi.org/10.5281/zenodo.17775713. Placental eQTM data are not yet publicly available, but can be shared upon reasonable request to MFH and TE.

SEPAGES biospecimens are stored at Grenoble University Hospital (CHU-GA) biobank (bb-0033-00069). SEPAGES data are stored thanks to Inserm RE-CO-NAI platform funded by Commissariat Général à l’Investissement. The data analysed in the presented study are not publicly available as they are containing information that could compromise the research participant’s privacy or consent. However, they are available upon reasonable request. The code used to generate the results is available at https://github.com/lbroseus.

## Declaration of interests

We declare no competing interests.
