# Supplementary material for: Adverse and adaptive placental DNA methylation changes linking prenatal air pollution exposure to child lung function: findings from the SEPAGES cohort
Source: eBioMedicine. 2026 Jun 24;129:106344. doi: 10.1016/j.ebiom.2026.106344 (PMC13320500; doi:10.1016/j.ebiom.2026.106344)

Content

Supplementary Figures:

Supplementary Figure S1. Flow chart of lung function measurements

Supplementary Figure S2. Distributions of air pollutant exposure levels

Supplementary Figure S3. Spearman correlation between air pollutant exposure levels

Supplementary Figure S4. Spearman correlation between lung function parameters

Supplementary Figure S5. Cell type proportions across air pollutant levels

Supplementary Figure S6. Association between air pollutants and lung function parameters

Supplementary Figures S7 A-B. Overlap between AMRs across lung function parameters

Supplementary Figure S1. Flow chart of lung function measurements

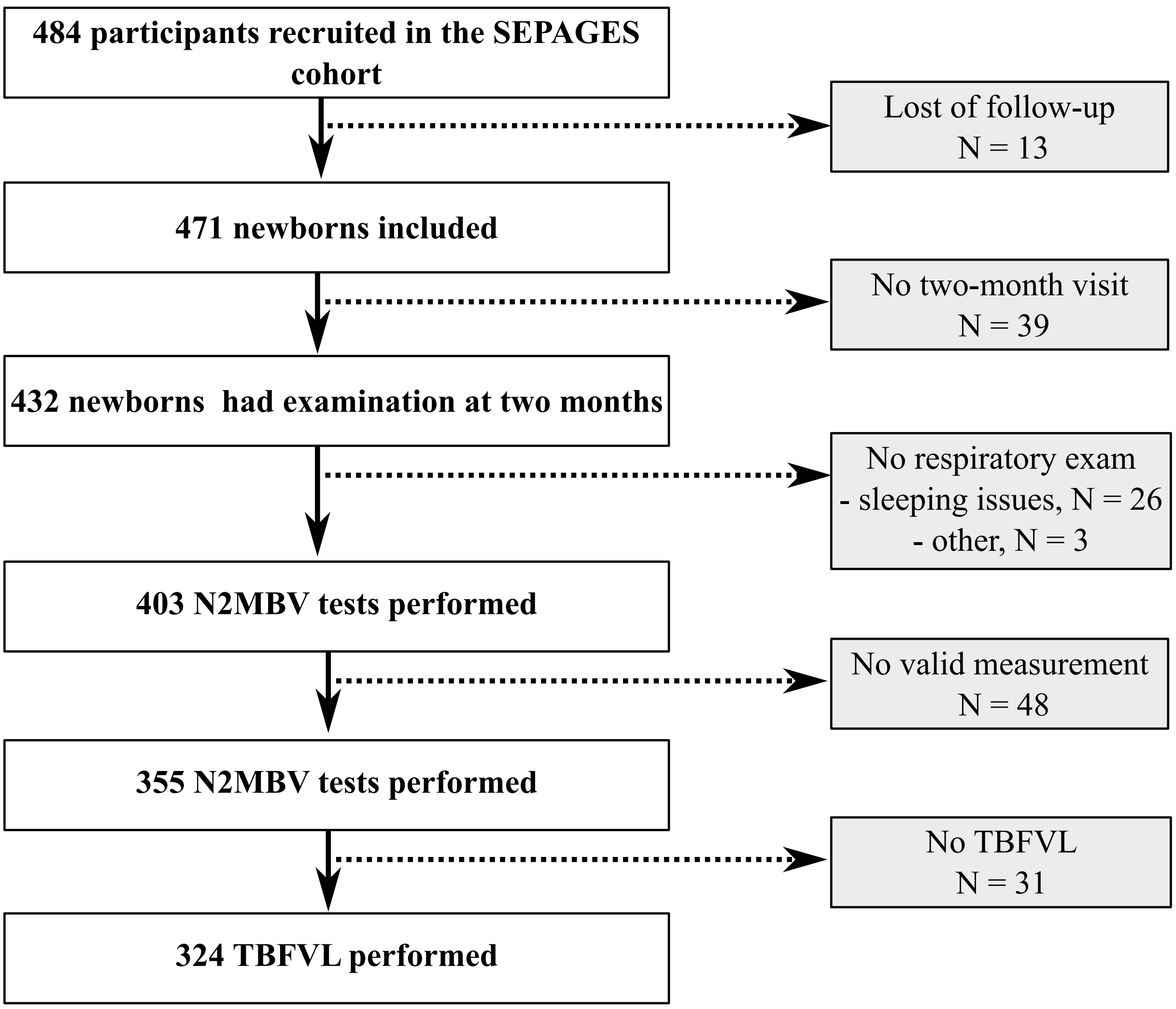

Supplementary Figure S2. Distributions of air pollutant exposure levels

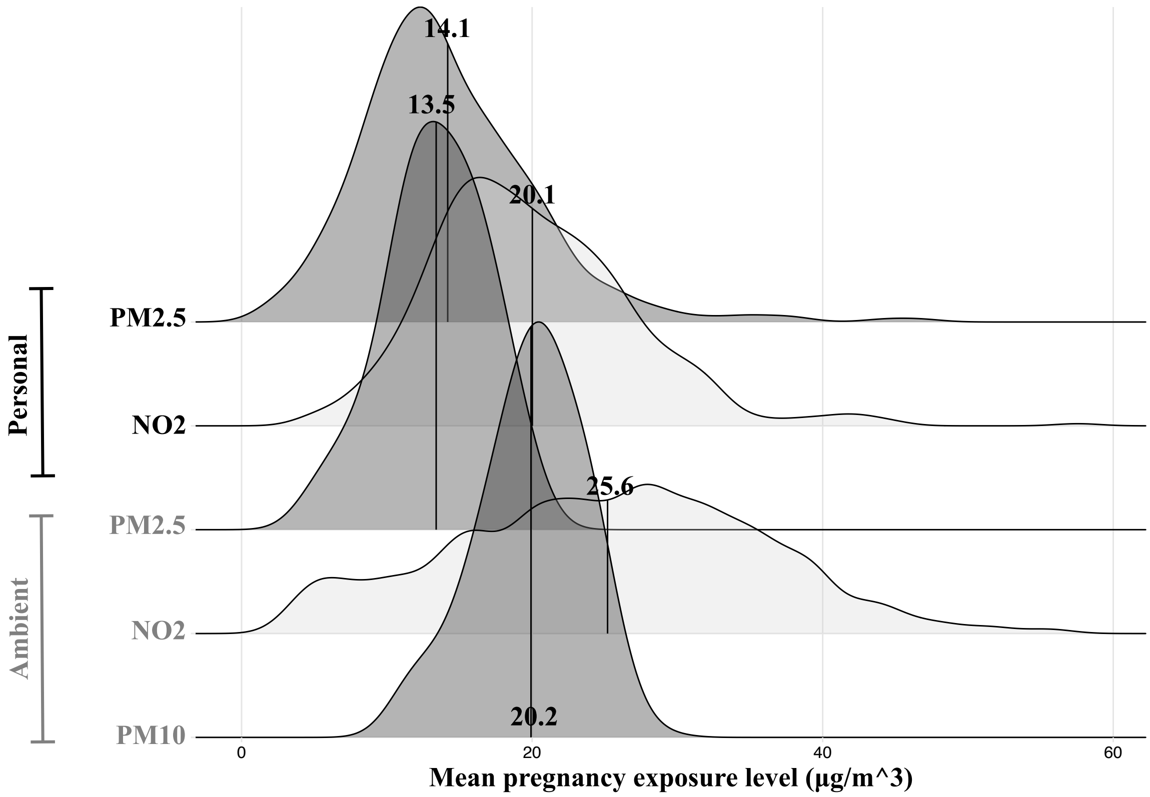

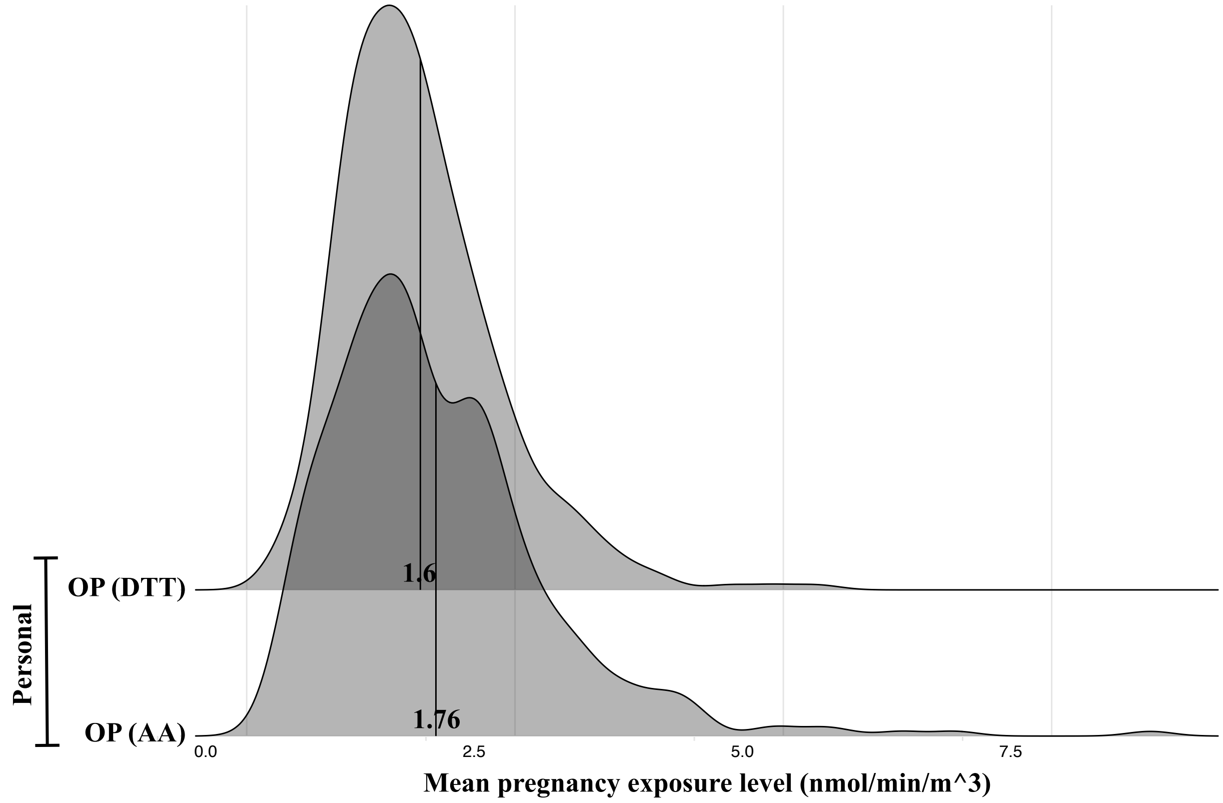

Supplementary Figure S3. Spearman correlation between air pollutant exposure levels

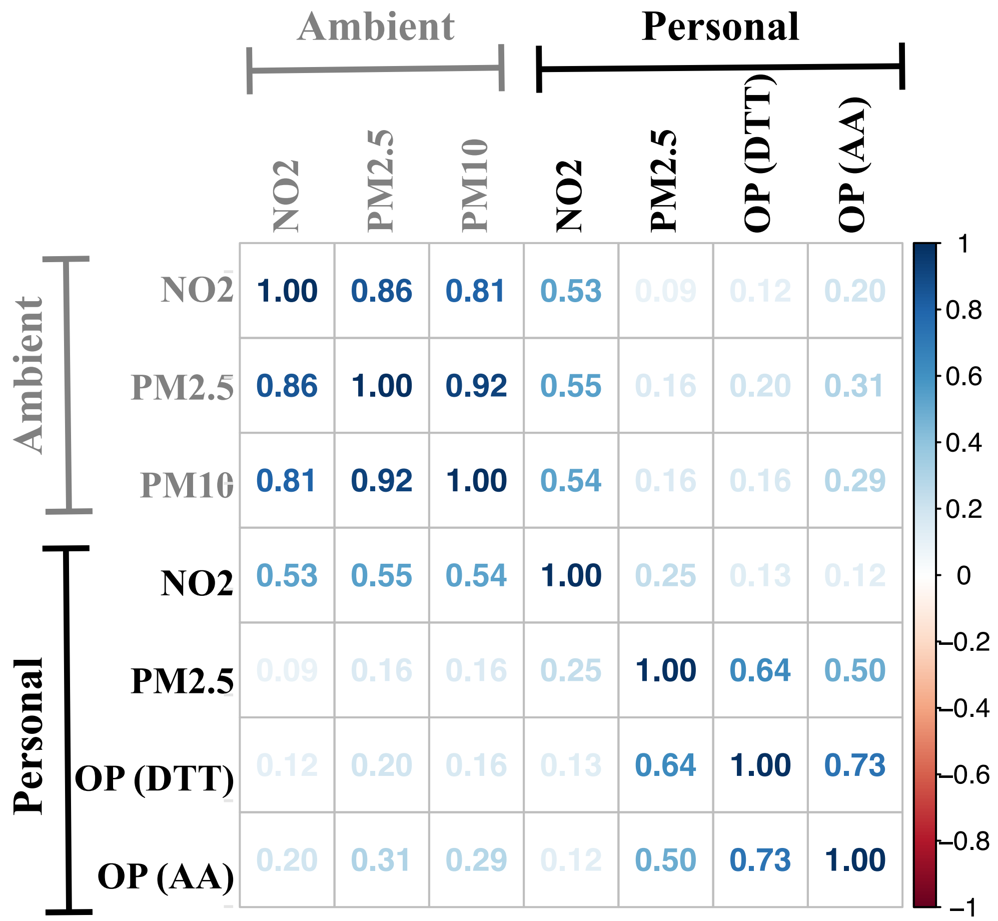

Supplementary Figure S4. Spearman correlation between lung function parameters

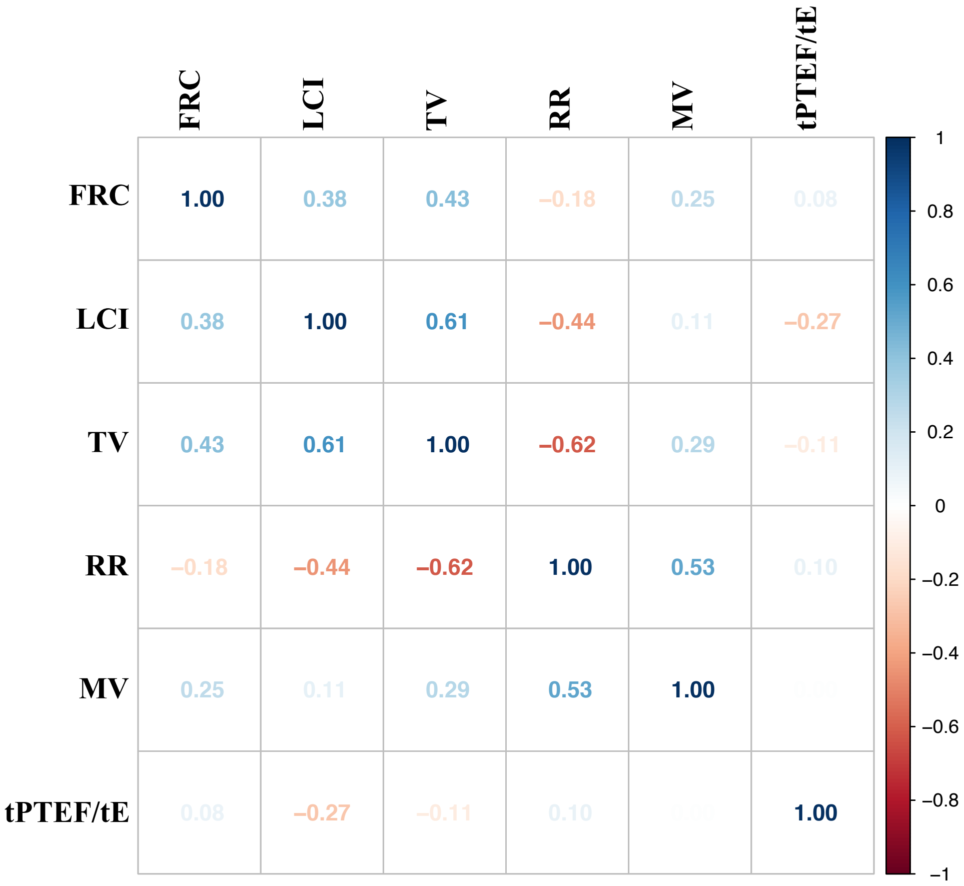

Supplementary Figure S5. Cell type proportions across air pollutant levels. Reference cell type proportions were estimated using the R package planet. We found not statistically significant association (nominal p-value<0.05; Wald test) between cell type proportions and exposure levels based on univariate regression models.

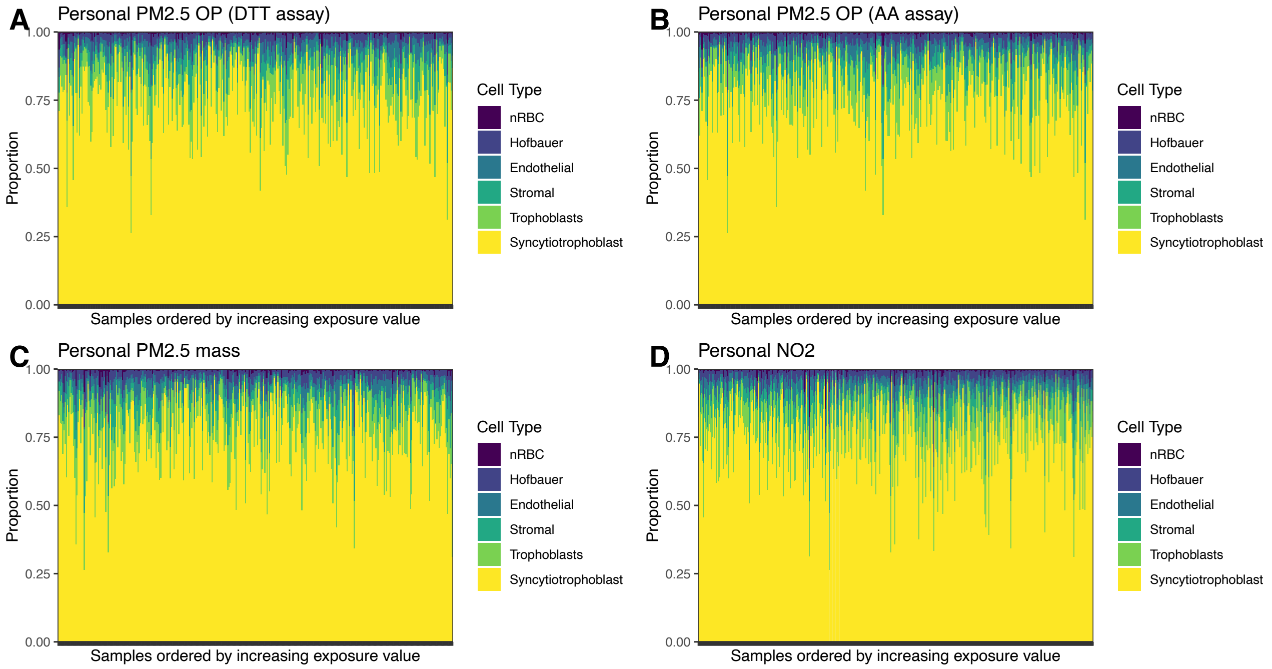

Supplementary Figure S6. Association between air pollutants exposure and lung function parameters. The association between each pair of air pollutant and lung function parameter was tested separately based on a linear regression model adjusted for potential confounders. Standardized effects sizes are displayed along with the standardized 95% confidence intervals.

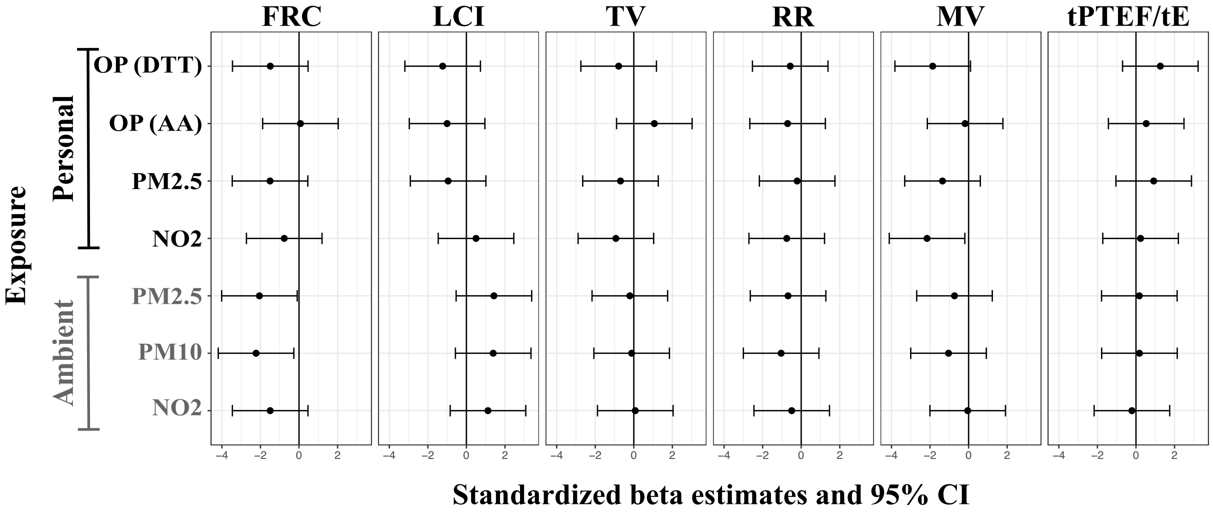

Supplementary Figure S7 A-B. Overlap between AMRs across lung function parameters.

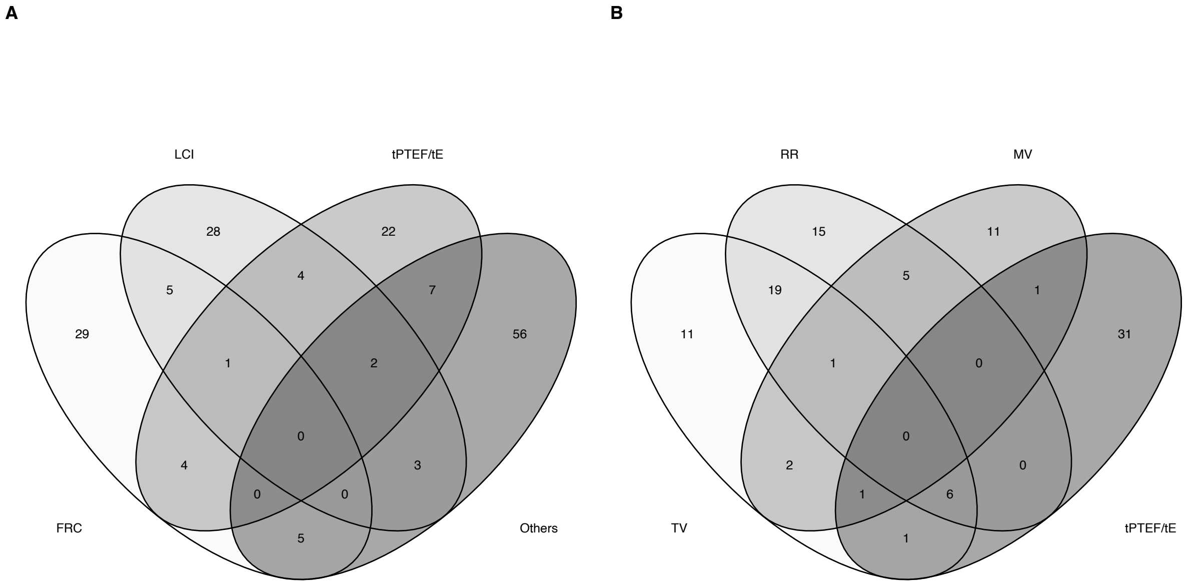

Supplement: Supplementary Figs. S1–S7 [file mmc1.pdf]
